# Study protocol for a zinc intervention in the elderly for prevention of pneumonia, a randomized, placebo-controlled, double-blind clinical pilot trial

**DOI:** 10.3389/fnut.2024.1356594

**Published:** 2024-02-21

**Authors:** Edwin F. Ortega, Dayong Wu, Weimin Guo, Simin Nikbin Meydani, Alexander Panda

**Affiliations:** Nutritional Immunology Laboratory, Jean Mayer USDA Human Nutrition Research Center on Aging, Tufts University, Boston, MA, United States

**Keywords:** zinc, pneumonia, intracellular immune cell zinc, older adults, nursing home residents, immunity, study protocol, COVID-19

## Abstract

Pneumonia is a major public health problem for older adults, being one of the leading causes of hospitalization and death, particularly for elderly nursing home residents. We previously conducted a clinical trial in which we demonstrated that 29% of nursing home residents had low serum zinc levels coinciding with a two-fold increase in pneumonia incidence and duration in comparison to individuals with adequate serum zinc levels. However, causality could not be inferred and necessitates a double-blind clinical trial. To determine the appropriate supplementation dose for such a trial we are conducting a randomized, placebo-controlled, double-blind clinical pilot trial aimed at delineating the optimal dosage (30 and 60 mg/day elemental Zn) and establishing safety. The results from the pilot study will be leveraged to inform our larger randomized clinical trial designed to study the effect of zinc supplementation in nursing home elderly with low serum zinc levels on respiratory infections, antibiotic use, and duration of sick days with pneumonia. In tandem with dose optimization, we will evaluate the correlation between serum zinc and pan-T cell zinc levels, given that T cells and their zinc levels are important in the response and resolution of respiratory infections but whose correlation has only been extrapolated and not demonstrated. Herein we present the study rationale and protocol, as well as discuss specific challenges we encountered in securing a manufacturer for the study agents and when recruiting from nursing home populations during the COVID-19 pandemic. In light of these experiences, we provide recommendations for future clinical trials under circumstances where supply chains are disrupted, and recruitment pools are constrained or unavailable.

**Clinical trial registration:**

https://clinicaltrials.gov/, NCT05527899.

## 1 Introduction

Pneumonia (PNA) poses a significant public health challenge, particularly for older individuals residing in nursing homes (NH) (1). In comparison to independently living older adults, NH residents have a higher frequency of infections, with PNA being the leading cause of infection-related hospitalization (2–4). Recent data points to a concerning trend of increasing incidence and mortality from PNA in older individuals (5). The annual incidence of PNA for older adults (>65 y) residing in the community is estimated at between 25 and 44 per 1,000 persons (4), whereas in long-term care facilities the incidence ranges from 99 to 912 episodes per 1,000 persons, with a median of 365 per 1,000 persons, a tenfold difference in incidence (1). When combined with influenza, PNA becomes the 5th leading cause of death in elderly individuals aged 65 to 74 y and the death rate from these infections increases exponentially above the age of 55 y (3). The economic impact of PNA is high, with treatment costs of $480/case in NH, and the cost for hospitalization exceeding $7,000/case (6). Therefore, it is imperative to implement effective strategies to prevent PNA in older adults.

One important contributing factor to the higher incidence of PNA in older adults is the age-associated decline in T cell function and the naïve T cell pool (7, 8). Intriguingly, there are striking similarities between zinc (Zn) deficiency and age-induced immunological changes (9, 10). In both cases, T cell defects have been identified as key contributors to higher susceptibility, morbidity, and mortality from infectious diseases, including PNA (9, 11–16). Previously, we reported that that 29% of NH residents in and around the Boston area had low serum Zn levels (<70 mg/dL), which was associated with significantly higher incidence and duration of PNA, increased antibiotic use, and overall mortality (17). Importantly, these associations were independent of age, BMI, albumin levels, other micronutrient levels, C-reactive protein (CRP), and other predisposing diseases for PNA.

Many studies have demonstrated that Zn supplementation enhances T cell-mediated function in Zn-deficient individuals, such as the elderly. Preliminary findings from our randomized, double-blind, placebo-controlled trial revealed that supplementation of elderly individuals with low serum Zn levels with 30 mg/d of Zn gluconate for 3 months improved serum Zn levels in majority but not all of the older adults (18). Specifically, 42% of those who completed the study in the Zn group did not reach adequate serum Zn concentrations (≥70 μg/dL) by month 3; these participants had very low serum Zn levels at baseline, with a mean ± SD of 53 ± 6 μg/dL and a range of 44–60 μg/d. Adjustment to increased Zn intake in highly Zn-deficient participants may require higher level of Zn supplementation or longer than 3 month supplementation period. Regardless, in this study we observed on average a significant increase in the percent of CD4+ T cells, and better maintained T cell function. A significant correlation between serum Zn level and T cell function, as assessed by their ability to proliferate, was also observed.

While our observational findings are supported by a small study conducted by Prasad et al. in which older adults supplemented with 45 mg/d of Zn for 12 months exhibited lower incidence of all infections including RI (19), there is an urgent need to demonstrate the efficacy of supplemental Zn to prevent pneumonia in older adults through a prospective clinical trial of adequate size and duration. As the first step toward that goal, we are conducting a 1-year double blind, placebo-controlled study to determine the effective and safe Zn supplementation dosage for NH residents. This dose will subsequently be used in a larger clinical trial to determine the effect of Zn supplementation in elderly NH patients with low serum Zn levels on the incidence of and morbidity from PNA. Additionally, results from a recent meta-analysis (20) indicated that Zn supplementation reduces the length of hospitalization in children with respiratory diseases. This further underscores the potential impact of Zn on respiratory health across different age groups.

Furthermore, while serum Zn levels serve as a reliable marker of Zn status at the population level and have been demonstrative to be responsive to supplementation, there is controversy about their accuracy in reflecting cellular Zn levels, which are important for optimal immune cell function, at the individual level. Since intracellular Zn levels are higher than serum levels, it is plausible that the serum Zn level may give an incomplete picture of cellular Zn state of an individual. Consequently, even in the presence of cellular Zn deficiency, serum Zn levels might fall within the lower end of the normal range. Therefore, as part of our primary objective, this pilot study will also evaluate how well serum Zn levels reflect cellular labile Zn levels prior to- and post-Zn supplementation. The findings of this proposal are scientifically essential, yet also sufficient, to make definitive decisions that inform the final design of our planned clinical trial.

## 2 Methods and analysis

### 2.1 Design

We are conducting a one-year long RCT to determine an effective and safe Zn supplementation dosage for elderly NH residents with low serum Zn levels (< 70 μg/dL), this threshold is recommended by Institute of Medicine, Panel on micronutrients (21) and others (22). Two Zn dosages, 30 or 60 mg/d, will be tested. The primary objectives are to establish the optimal dose of Zn supplementation required to achieve adequate serum Zn levels and estimate the correlation between serum and intracellular labile Zn levels. The secondary objectives are to assess adverse events and immune outcomes and collect data on PNA to design a larger clinical trial effectively. The selection of the dose will be determined by the combination of time and dosage that exceeds threshold for achieving adequate serum Zn levels in the majority of subjects (>90%) while remains below designated threshold for incidence of the side effects (<10% moderate or severe side effects, see below).

**Figure 1:**
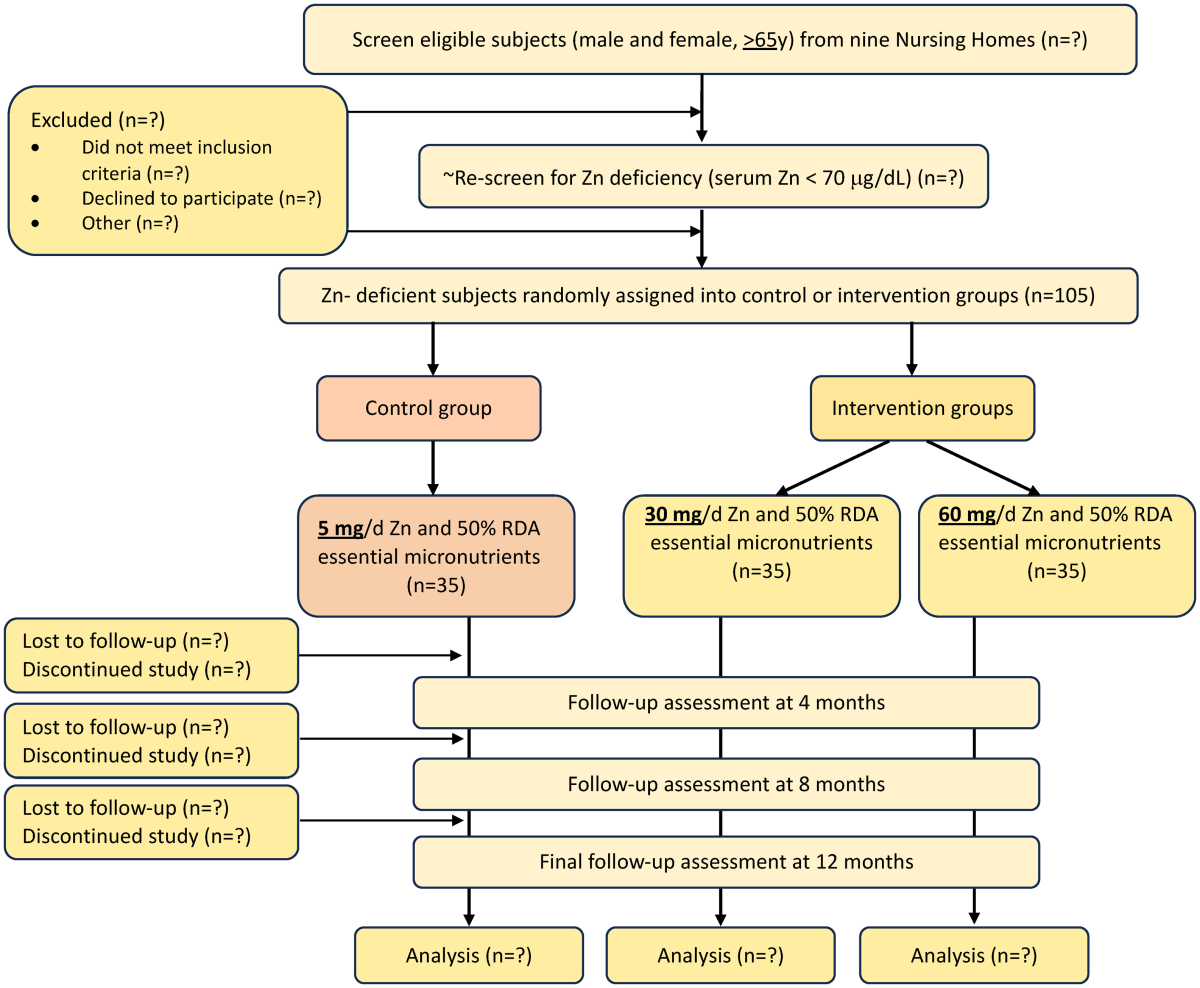
Zinc intervention in the elderly for prevention of pneumonia study flow diagram.

A total of 105 male and female elderly subjects (≥65 y) with low serum Zn (<70 μg/dL) will be enrolled from a NH within 1 h driving distance from Boston (from an estimated 16 NHs, ~900 residents) and be assigned to one of the 3 groups. Group 1 will serve as control and will receive a capsule containing ½ Recommended Dietary Allowance (RDA) of all the essential micronutrients (MN), including 5 mg of elemental Zn (1/2 RDA MN); group 2 will receive a capsule containing ½ RDA MN including 30 mg/d elemental Zn, and group 3 will receive a capsule containing ½ RDA MN including 60 mg/d elemental Zn. Zn is provided as Zn gluconate. The overall study design and number of subjects is shown in Figure 1 and the frequency of data collection is shown in Table 1.

**Table 1:** Zinc intervention in the elderly for prevention of pneumonia study outcomes and collection frequency.

	Age	Sex	Race	Ethnicity	SES^1^	Supplement use (Ca, Vit D, Fe)	Smoking status	Alcohol status	Disease status	Prescribed medication	Serum & intra-cellular Zn	T cell profile	Cu, Fe, Ferritin, CBC^2^, albumin, CRP^3^, BMI^4^
Baseline	x	x	x	x	x	x	x	x	x	x	x	x	x
Month 4											x	x	x
Month 8											x	x	x
Month 12											x	x	x

^1^SES, Socioeconomic status. ^2^CBC, Complete blood count with differential. ^3^CRP, C-reactive protein. ^4^BMI, Body mass index. Total blood collected 120 mL. Other outcomes: Every 2 weeks: adverse event assessment- screen and record NH residents every 2 weeks for the following symptoms: nausea, vomiting, abdominal discomfort, neurologic abnormalities. Throughout the study: new disease, medications, transfers.

We will use each NH unit’s dose dispensing system to deliver the capsules as in our previous NH Study (23). In that study, 98% of subjects consumed the capsule for more than 90% of the 1-y supplementation period. Adherence will be checked by measurement of Zn from blood samples collected every 4 months (Table 1).

### 2.2 Study population and eligibility criteria

Study participants will be long-stay elderly NH residents (≥65 y). Long-stay residents of NH are at high risk of PNA and have higher morbidity and mortality due to PNA (3). Our previous study showed that Zn deficiency is prevalent in NH residents (29%), and those with low Zn had higher incidence and morbidity from PNA (17). In addition, the need for a year-long study to capture seasonal variation in infection, for careful documentation of infections, and to keep as many as dietary and environmental factors constant in our future randomized clinical trial, makes the NH setting preferable.

#### 2.2.1 Inclusion criteria

The inclusion criteria for this study are: older individuals (≥ 65 y), with a > 6-month life expectancy (as judged by the physician), who are willing to be randomized into the 3 study groups, can swallow pills, are not currently on antibiotics, willing to receive influenza vaccine, and have a BMI > 18 kg/m^2^ and albumin >3.0 g/dL (Table 2). If participant is consuming RDA levels of supplement, they must be willing to replace their supplement with our control supplement. Calcium, vitamin D, and iron supplements are permitted.

**Table 2:** Exclusionary and inclusionary criteria.

Inclusion criteria	Exclusion criteria
Elderly males and females (>65 years) >6-month life expectancy, as judged by physician Willing to be randomized into study groups Able to swallow pills Not currently on antibiotics If consuming RDA levels of supplement, willing to replace with our control supplement; calcium, vitamin D and iron supplements are permitted Willing to receive influenza vaccine BMI > 18 kg/m ^2^ and albumin >3.0 g/dL	Anticipated transfer or discharge within 3 months of enrollment Bed- or room-bound continuously for previous 3 months Presence of lung neoplastic diseases or other neoplastic diseases requiring chemotherapy and/or immunosuppressive drugs (including ≤10 mg/day prednisone) Nasogastric or other tube feeding Long-term (≥30 days) IV or urethral catheters Presence of tracheostomy or chronically ventilator-dependent Chronic prophylactic antibiotic treatment or long-term antibiotics Those with PEM defined as albumin <3.0 g/dL and BMI <18 kg/m^2^ Consumption of supplements containing more than the RDA level of nutrients known to affect the immune response, i.e., vitamins E, C, B6, selenium, Zn, or β-carotene Diagnosis of PNA or other infection at baseline will not exclude a subject, but will postpone enrollment to 4 wks after PNA symptoms have cleared

#### 2.2.2 Exclusion criteria

Our selection of long-term NH elderly and exclusion criteria (Table 2) will eliminate most comorbid conditions that could overwhelm contributions of the immune system and thus the effect of our intervention. Exclusionary criteria includes: anticipated transfer or discharge within 3-months of enrollment, bed- or room-bound continuous for the previous 3-months; presence of lung neoplastic diseases or other active neoplastic diseases requiring chemotherapy and/or immunosuppressive drugs (including ≤10 mg/day prednisone); nasogastric or tube-feeding; long-term (≥ 30 days) IV or urethral catheters; presence of tracheostomy or chronically ventilator-dependent; chronic prophylactic antibiotic treatment or long-term antibiotics; those with PEM defined as albumin <3.0 g/dL and BMI < 18 kg/m^2^; consumption of supplements containing more than the RDA level of nutrients known to affect immune responses, i.e., vitamin E, C, B6, selenium, Zn, or β-carotene. Diagnosis of PNA or other infection at baseline will not exclude subject participation but will postpone enrollment to 4 wks after PNA symptoms have cleared.

### 2.3 Intervention

We are conducting a randomized, double-blind, and placebo-controlled trial, in which Zn deficient NH elderly (< 70 μg/dL) will be supplemented with a control (½ RDA of all micronutrients including 5 mg/d of Zn) or ½ RDA of micronutrients with total of 30 or 60 mg/ of elemental Zn for 12 months.

### 2.4 Outcomes

#### 2.4.1 Primary outcomes

The primary outcomes of this study are twofold: to identify an effective and safe Zn supplementation dosage for NH residents as well as to investigate the correlation between serum and cellular T cell or neutrophil Zn levels. Blood will be collected using appropriate Zn-free syringes, gloves, and tubes. Serum Zn concentration will be measured as described (24, 25). To quantify labile Zn in neutrophils RBC’s will initially be removed from whole blood using an RBC lysis buffer (eBioscience) compatible with flow cytometric analysis and minimally impacting lymphocytes. For assessment of labile Zn in pan-T cells, PBMC’s will be isolated using SepMate PBMC isolation tubes following the manufacturer’s instructions. Labile Zn in both pan-T cells and neutrophils will be determined as previously reported (26). Briefly, each resulting cell suspension, containing an estimated 2 × 10^6^ cells, will be loaded with 1 μM FluoZin3-AM ester and incubated with 2 μM N, N, N′, N′- tetrakis-(2 pyridylmethyl) ethylenediamine (TPEN, for minimum value or F_min_) or 100 μM Zn sulfate and 50 μM pyrithione (for maximum value or F_max_). The cells will then be stained with fluorescence labeled anti-CD3 (T cells) or anti-CD16b (neutrophil) antibody. Pan-T cells will be analyzed in a flow cytometer for the fluorescence intensity of FluoZin-3 in FL-1 and anti-CD3 in FL-4, while neutrophils will undergo flow cytometric analysis for fluorescence intensity of FluoZin-3 in FL-1 and anti-CD16b in the FL-4 channel.

#### 2.4.2 Secondary outcomes

The secondary outcomes of this study are: (1) T cell number and phenotype, (2) T cell proliferation, (3) PNA incidence, duration, hospitalization, and antibiotic use, (4) number of adverse events including gastrointestinal symptoms (such as nausea, vomiting, abdominal pain), and (5) Cu levels. We will also measure white blood cell count and differential, clinical chemistry for assessment of anemia, general health, and CRP and albumin level for any correction needed for serum Zn level. Fe and Ferritin will be assessed to determine if any anemia observed is due to copper (Cu) and not Fe. At baseline, age, sex, ethnicity, smoking and alcohol history, past and present supplement use, weight, BMI, diseases, medication use, transfer, and hospitalization will be recorded. In addition, new diagnosis, and medication will be recorded throughout the study.

Total pan-T cells, helper T cells (CD3+), and cytotoxic T cells (CD8+) in PBMC’s will be determined by measuring populations of CD3+, CD4+, and CD8+ cells, respectively, using a flow cytometry method previously described in our Zn supplementation study (18). T cell proliferation, a measure of function, will be assessed by incubating PBMC’s in the presence of T cell mitogens Concanavalin A (Con A) or Phytohemagglutinin (PHA), or antibodies against CD3 (T cell receptor) and CD28 (T cell co-receptor) for 72 h. Cultures will be pulsed with 0.5 μCi [3H]-thymidine (Perkin Elmer) during the last 4 h of incubation and then harvested onto glass-fiber mats (Wallac) with a Perkin Elmer cell harvester (Perkin Elmer). Cell proliferation will be assessed by the amount of [3H]-thymidine incorporated into the DNA as determined with the liquid-scintillation counting with a Micro Beta 2 MicroPlate counter (Perkin Elmer).

While the study is not powered to determine incidence of PNA, we will document PNA incidence to gain insight into any logistical issues and, if needed, adjust sample size for the clinical trial. We will diagnose PNA using criteria established by the Infectious Diseases Society of America/American Thoracic Society IDSA/ATS on community acquired pneumonias (27). In this pilot trial sputum cultures and chest X rays will only be obtained by the discretion of the care-taking doctor. The study physicians will collect data from the subject examinations, interviews, and record reviews to determine incidence and duration of PNA. Symptoms can include cough with or without sputum production, chest pain, dyspnea, and fever. Signs of infection include elevated temperature (≥38°C), tachycardia, tachypnea, abnormal breath sounds, and dullness to percussion of the chest. The diagnosis requires radiological findings of 1 or more new pulmonary infiltrates. An infection is considered resolved when all symptoms cease. A new infection is defined as one occurring after ≥7 symptom-free days. These procedures were successfully used in our previous study (17). We will visit each individual NH routinely every 2 weeks and perform a chart review and a focused physical exam (lung for PNA and GI, for adverse events) on all study participants. While not a secondary outcome of the study, upper and other lower RIs will be documented using procedures described before (17).

We will screen and record NH residents every 2 weeks for the following symptoms/adverse events: (1) Minor side effects: Nausea, and bad taste. These side effects were relatively common in studies using doses well above 60 mg/day. If patients are willing to continue taking Zn despite these minor side effects, we will continue Zn supplementation. (2) Moderate side effects: Nausea, vomiting, stomach pain, diarrhea. These adverse events are extremely rare at doses of 30 and 60 mg/day. These will be designated as not acceptable, and participants will be dropped from the study. (3) Severe side effects: Flu-like symptoms (fever, chills, cough, headache), neurologic symptoms such as paresthesia, gait disturbance or photophobia. These adverse events have not been reported at doses of 30 and 60 mg/d. Participants exhibiting these adverse events will be dropped from the study. We will record the number of side effects and their duration throughout the 12-months duration of the study for both Zn doses. If treatment stops due to adverse events this event will be counted as such, and participant will be dropped from study. If at any point more than 10% of planned enrollments have experienced at least 1 moderate or severe side effect, we will close this study arm.

Clinical chemistry profile, albumin, Cu, Fe, Ferritin, CRP, and complete blood count with differential measures (CBC-diff) will be analyzed on all subjects as previously described in our Zn supplementation study (17, 18). Importantly, intestinal absorption of Cu is inhibited by Zn. Thus, chronic intake of high doses of Zn may be associated with Cu deficiency (28). This concern is the main basis for the upper tolerable limit (UL) for Zn (21). However, notably, this UL refers to chronic intake of Zn in subjects with adequate Zn level and does not preclude the use of higher doses of Zn for treatment of deficiency or diarrhea. The presence of 0.8 mg Cu in our placebo and Zn capsules will ensure no adverse effect on Cu status. Further, no serious adverse effects have been reported for long-term (up to 5 yrs) intake of up to 80 mg of Zn/day in older adults (29), who had baseline serum levels higher than those proposed in this study. Blood will be examined with a Baker 9,000 Hematology Analyzer for RBC and WBC counts and a blood smear will be used to estimate WBC differentials. This information will be used for statistical adjustment by analysis of co-variance and/or for assessment of side effects. CRP is included as it is a marker of inflammation which may impact Zn levels transiently and will be used for statistical adjustments as needed. BMI will be calculated from baseline weight and standing or knee height (in subjects who cannot stand or are contracted) (30).

### 2.5 Sample size

We plan to enroll 105 study participants (*n* = 35/group). Power is based on 27 study participants/group, allowing up to 20% attrition over the 1-year supplementation period. Preliminary data from Zn supplementation of 30 mg/day reported 58% conversion of study participants (17). We anticipate at least a 90% conversion for the 60 mg/d dose. In order to detect an increase in percent conversion from 58 to 90%, with 80% power and type I level of alpha = 0.05, 27 study participants per treatment group are required. Sample size is determined based on testing for a difference in percent conversion between the two dose groups using a one-sided Fisher’s Exact test (31).

### 2.6 Recruitment

A total of 105 residents will be enrolled from an estimated 900 elderly NH residents (Figure 1). We have support from 16 NHs (~1,600 residents) located within 1 h driving distance from Boston. Based on our previous experience (23, 32) we estimate that 900 NH residents need to be screened to identify 350 (Figure 1), who will meet our eligibility criteria (Table 2) who will then be screened for Zn and albumin levels to identify 105 subjects with low serum Zn levels. Although individual NH populations are heterogeneous, when similar methods for collection of information and time intervals are used, the comparative incidence of RI and PNA is quite similar. Our data also demonstrated (17) similar prevalence of low Zn levels in all NHs we studied in the Boston area, regardless of socioeconomic status, ethnicity, or NH affiliation (academic or not, or VA). Considerations for choosing participating NHs will include willingness to participate in the study, compliance with all state Department of Public Health regulations, number of beds (>50), compliance with state staffing requirements and sick days, and absence of shared staff between different units in multi-unit facilities (33).

### 2.7 Randomization, allocation concealment, and blinding

Subjects will be assigned to each arm via block randomization with a block size of 6. The randomization will be done via the built-in random number generator included in the study database and data capture system, REDCap. Placebo and Zn supplements will be provided in identical capsules to maintain double blinding. The database manager will retain the randomization code. Other members of the study team, including the Co-PIs, will be blinded to treatment assignments. The placebo and Zn capsules have been developed to have identical appearance to ensure blinding of the treatments.

### 2.8 Data collection and management

Clinical measures and study data collected on-site will be captured by the study staff using Electronic Case Report forms (eCRFs) developed in REDCap, a Research Electronic Data Capture platform hosted at the Human Nutrition Research Center on Aging at Tufts University (HNRCA) and secure study dedicated laptop. These eCRF forms are accessible via the web or an iPad app developed for use in instances where internet access is limited or lacking. In addition to standard clinical and anthropometric measures, these eCRFs will also include health status and intervention response questionnaires to assess response to intervention. REDCap is a secure HIPAA- and 21 CFR 11-compliant web-based application for building and managing online surveys and databases that are structured to support data capture for multisite clinical trials. It provides real-time data entry validation and a de-identified export mechanism to common statistical packages. Ongoing review of entered data will occur and incomplete data adjudicated within 1 week of entry. In all instances, access to the server will be restricted to data management staff at Tufts University.

Designated users from individual sites will have access for data entry, along with read access to individual site data, subject randomization, data dictionaries and collection forms. Laboratory data will be exported and be processed using Microsoft Excel and reviewed for outliers using standard statistical software (SAS). Laboratory data will be stored and archived on secure servers at the HNRCA with scheduled daily back-ups. Upon completion of the study, all data will be de-identified and converted to/archived in csv files for long-term storage and sharing. The files will contain variables with standardized naming conventions consisting of prefix, root and suffix system and include variable description labels. All data and corresponding documentation will be preserved in perpetuity and related files in the different formats will be linked by file naming conventions and revision date. Corresponding documentation including protocols, value ranges, formats and data description will be developed and stored with the data files. Adherence to the data management plan will be monitored and reviewed monthly. Implementation of the plan and progress on the data collection, archiving, and sharing will be included in reports.

### 2.9 Statistical analysis

This pilot study is a randomized dose-finding trial with the primary objective to establish optimal dose of Zn supplementation to achieve adequate serum Zn levels and estimate association between serum and cellular Zn levels. The effectiveness will be assessed based on the ability to increase serum Zn levels to adequate levels in the majority of participants (>90%) and in the shortest duration of supplementation and at the same time results in <10% of moderate or severe adverse events. Subjects will be deemed Zn adequate on the first occasion where their Zn serum level reaches ≥70 μg/dL. Subjects will not be included in the calculation of this proportion if they discontinue supplementation before reaching Zn-adequate status. Pearson correlation will be estimated between serum and cellular Zn levels. Violations of linearity will be assessed graphically, and sample product moment correlation method (34) will be used to produce two-sided confidence intervals for the estimated correlation. Analysis among dose groups will follow an intention-to-treat approach. Comparison of proportion of Zn adequate between dose groups will be tested using Fisher’s exact test with risk ratios at each time point. Continuous outcomes that have repeated measures taken at baseline, 4, 8, and 12 months will be analyzed with mixed effects linear regression which considers within-subject correlation of outcomes. We will assume linear time trends and test the dose effect by the treatment by time interaction term. Assumptions of normality and homoscedasticity will be evaluated in these models and appropriate variable transformations will be applied if needed. Age, sex, and NH site will be included in the models as covariates. A significance level of 0.05 will be used for each secondary outcome. To control for multiple testing across groups, the type I error will be reserved for the comparison between the placebo group and the selected Zn dose group. Summary statistics on any missing data will be reported, including types and patterns of missingness. Subjects with partial outcomes over time can be included in the model fit since the mixed-effects linear modeling framework allows inclusion of subjects having different numbers of repeated measures. Multiple imputation based on the multivariate normal model will be used in the case of missing covariates. Analysis will be performed using SAS (version 9.4; SAS Institute, Cary, NC).

## 3 Discussion

### 3.1 Rationale for composition of treatment capsules

Based on our pilot study and review of the literature, 30 mg/d Zn is effective and safe. However, 30 mg/d was effective in improving serum Zn level to adequate in only 58% of Zn deficient participants. Further, a significant correlation between the change in Zn level and T cell proliferation was observed. We concluded that either a longer duration of supplementation with 30 mg/d or higher doses of Zn will be needed to effectively increase Zn serum to adequate levels in NH residents. Our study design will allow us to determine if a longer period of supplementation with 30 mg/d will be effective in improving Zn status in most if not all NH elderly. However, since longer than 3-months supplementation with 30 mg/d may not raise serum levels adequately, we propose to use a higher dose (60 mg/d) as well. Our rationale for selecting 60 mg/d is as follows: (1) Prasad et al. supplemented older subjects with average serum Zn level of 90 ug/dl for 1 year with 45 mg/d of Zn and noted, on average, ~12 ug/dl change in serum Zn level (13). Since we also observed about 12 ug/dl increase with 30 mg/d supplementation, yet 42% of subjects remained Zn deficient, we propose that a higher than 45 mg/d will be needed to improve Zn status in Zn-deficient elderly. (2) 60 mg/ d of Zn is a high, yet safe level of Zn for this population.

### 3.2 Rationale for using ½ RDA of multivitamins as placebo

Our rationale for using ½ RDA of multivitamins as placebo is that NH residents have a heterogeneous multivitamin intake. Less than 2/3 RDA intakes of B vitamins, Vit D, and Zn have been reported (35, 36). Some of these nutrients have been shown to be required for proper function of the immune system. To reduce the possible variability introduced by low intakes of multivitamins, and because we are requesting that the participants discontinue their multivitamin supplements, we believe using 50% of RDA for essential nutrients including 5 mg Zn as a control placebo is justifiable. We have not proposed to use RDA levels of multivitamin because that will bring the total intake of the multivitamin to above the RDA; some of these nutrients have been shown to enhance the immune response at above RDA level and thus might attenuate the independent effect of Zn. We expect that the older individuals selected by our criteria will have intakes that are <50% of the RDA of multivitamin (23, 37). Thus, providing them with 50% of the RDA level of these nutrients will bring their total intake to at least the RDA level.

### 3.3 Rationale for selected primary outcomes

Zn status can be assessed by measurement of Zn in plasma, erythrocytes, neutrophils, lymphocytes, and hair. Measurement of Zn in the plasma is simple and readily available in many laboratories. A low plasma Zn usually is defined as a value less than 70 ug/dL (22, 38). Because most plasma Zn is bound to albumin, measured Zn levels will typically be reduced in patients with hypoalbuminemia. However, in clinical practice, it is not particularly helpful to correct measured Zn levels for hypoalbuminemia, because plasma levels are only loosely correlated with Zn stores, and because Zn replacement is usually provided to patients with low Zn levels, regardless of albumin status, particularly in the context of chronic disease. Nevertheless, we are measuring plasma albumin levels to adjust if needed. In addition, inflammation and acute phase response can impact plasma Zn levels. Thus, we will measure CRP levels to exclude variation due to acute illness and inflammation.

Plasma (or serum) Zn concentration is the most widely used biomarker to determine Zn status. Plasma Zn concentrations normally respond to Zn supplementation, especially in subjects with a low or moderately low baseline (18, 39–41). Generally, the reduction of plasma Zn concentrations occurs when subjects are deprived of Zn (42–44). Measurement of serum Zn has recently been recommended by WHO, UNICEF, IAEA, and International Zn Nutrition Consultative Group (IZiNCG) as the best biomarker of Zn deficiency in a population and is widely used as such because “it responds as expected to dietary modifications and is associated with functional outcomes” (45, 46). Although serum Zn level serves as a reliable indicator of Zn status at population level and is responsive to supplementation, there is controversy concerning its representation of cellular labile and total Zn level (crucial for function) at the individual level. At present, measurement of the serum/plasma Zn level, and confirmation of a deficient state by administering a Zn load are considered to be the most reliable methods of diagnosing Zn deficiency. However, since the intracellular level of Zn is higher than its serum level, it is plausible that the serum Zn level may not faithfully reflect the cellular state of an individual. The criteria for Zn deficiency are decreased cellular Zn level in either lymphocytes (<50 μg/10^10^ cells) or granulocytes (<42 μg/10^10^cells) (47). Given the importance of Zn for T cell function, the key role of T cells in PNA, and the well demonstrated deterioration of T cell function with age, we will assess Zn status in T cells.

### 3.4 COVID-19 related challenges

The COVID-19 pandemic affected the initiation of clinical trials overall. Although an increase in the overall numbers of clinical trials could be observed both in Europe and the United States, the number of initiated non-COVID-19 trials was reduced, with a slightly larger relative decrease in the United States. Short-term trends will likely show a reversal of this trend after we have exited the pandemic phase.

Our study was initiated 18 months after the first case of the 2019 novel Coronavirus in the U.S. was detected on January 18, 2020. At this point in time cases were still increasing, even though vaccines were available, and multiple NH facilities in Massachusetts and nationwide reported local outbreaks. Most of the NH would not allow us to initiate screening even though we had secured prior commitments from medical and nursing directors. Here we benefited from relations established from our previous NH studies and from our reputation as trustworthy, responsible investigators.

Decisions for renewal and for new commitments were now made at the highest executive levels of the respective NH organizations. A lesson learned for future clinical trials is that commitments from NH should be obtained at the highest executive levels of NH organizations and the need for a contingency plan for periods of limited resources due to local or national public health emergencies should be addressed.

We as the investigators are aware of the potential risks, we may introduce unintentionally to the NH residents. Therefore, COVID19 testing prior to entering the NH facilities, face masks, the use of other personal protective equipment and hand hygiene will be and continue to be strictly implemented. All study team members are vaccinated for COVID19 and influenza.

Nurses and healthcare workers were under extreme stress leading to workforce shortages as well as increased health care worker burnout, exhaustion, and trauma. These pandemic-related challenges have taken place in a context of significant pre- existing workforce shortages and maldistribution, as well as in a workforce where burnout, stress, and mental health problems (including an ongoing risk of post-traumatic stress disorder) were already significant problems. To avoid additional tasks to nursing staff we will remind NH residents, family members, health care proxies and guardians to address any study related questions to the investigators directly. We will provide a cell phone number which could be dialed for 24 h any day during the week including weekends. During the initial phase of the study the study physician was present on site every day even if no screening, recruiting or follow up took place.

The COVID-19 pandemic has alerted businesses to the fragility of global supply chains. Heightened demand, trade restrictions, factory closures, rising freight rates and reliance on ‘just-in-time’ inventory systems have led to global shortages and inflation. In view of the emerging COVID-19 pandemic caused by SARS-CoV-2 virus, the search for potential protective and therapeutic antiviral strategies was (and is) of particular and urgent interest. Despite the lack of clinical data, certain indications suggested that modulation of Zn status may be beneficial in COVID-19. This triggered an incomparable demand in over-the-counter Zn supplements which exponentiated the national and global supply chain disruptions for Zn products. In the context of these supply chain disruptions our original Zn supplement supplier was no longer able to deliver the ordered Zn supplements and placebo pills. Thus, we had to search for alternative companies who were able to produce the Zn supplement including the placebo pills, as described, in a timely manner and at a reasonable price. We were successful in doing so only after contacting and negotiating with several companies. This delayed the timely start of the study significantly. The lesson learned here is that alternative companies should be at least identified besides the actual company contracted who will be capable of delivering the intervention drug or supplement in the event of an unexpected shortage.

## 4 Conclusion

In summary, this double-blind, placebo-controlled, randomized trial will rigorously test our formal hypotheses, providing valuable insights into the primary objectives concerning the safety and efficacy of Zn supplementation, as well as the correlation between serum and cellular Zn concentrations. The findings derived from this pilot study will generate evidence to inform the final design of our forthcoming larger clinical trial we intend to undertake. Ultimately, both this study and the subsequent comprehensive clinical trial will mark a pivotal step toward confirming Zn as a risk factor for PNA and developing a novel, simple, and cost-effective nutritional intervention to mitigate PNA risk and associated morbidity and mortality among NH residents. A nutritional intervention, once available, would significantly enhance the health and quality of life for the elderly population by changing the standard of care for at-risk elderly individuals with respect to PNA prevention. This would consequently yield substantial cost savings associated with the care of elderly NH residents. Further, given the complexity of clinical trials, particularly during a global pandemic, a contingency plan is essential to address the challenges with study resources, participants, manufacturers, and stakeholders.

## 5 Ethics and dissemination

### 5.1 Ethics statement

This study has been approved by the IRB at Tufts Medical Center and Tufts University Health Sciences Campus (IRB #: 1427). This study is being conducted in accordance with ICH GCP requirements. Protocol modifications are communicated to the Institutional Review Board at Tufts Medical Center and Tufts University Health Sciences Campus. This study is registered on ClinicalTrials.gov with the identifier: NCT05527899.

### 5.2 Consent or assent

We held in-service sessions at each participating site. Staff were given an overview of the study and procedures, eligibility requirements and the research team’s qualifications, and were assured that they will not be asked to help obtain informed consent (48) or to perform additional documentation other than aiding in the identification of eligible subjects, recording the daily supplement administration. Once the individual NH administrative approval was obtained, the medical directors and attending physicians of all participating homes were consulted to ensure that the protocol will not disrupt any medical care activities. Following each in-service, our research team and the nursing staff evaluated residents for eligibility. Then, each PCP was notified that their patients are eligible for the study. All recruitment materials were written clearly and in large enough print that would be easily read and understood by this audience. Descriptions of the tests was kept simplified to ensure understanding yet accurately reflect the procedure. Potential health benefits of the study were described, and the risks associated with participation were clearly disclosed. Several reports have focused on the problems associated with obtaining consent from NH residents or their proxies (49–51) We used these guidelines as we did in our previous NH study (23, 32) to implement ethical and efficient strategies to successfully obtain informed consent from subjects. All IRB approvals was obtained from all participating institutions and facilities.

### 5.3 Confidentiality

All information, data, documents, and materials related to the participants will be kept confidential. Any breach of confidentiality shall be reported immediately to the sponsor. The study PI, Co-Investigators, Study Coordinators, and Statistician will have access to participant identifying information, if needed. Otherwise only de-identified data will be shared with co-investigators. We will take reasonable measures to safeguard the confidentiality of the information, including maintaining secure storage, restricting access to authorized personnel, and implementing data encryption.

### 5.4 Dissemination

The findings from this research study will be submitted to and published in a peer-reviewed journal. We plan to leverage these data to apply for a clinical trial grant under National Heart, Blood and Lung Institute. Request for use of study data must be approved by the study sponsor and IRB.

## Author contributions

EO: Conceptualization, Writing – original draft, Writing – review & editing. DW: Conceptualization, Writing – review & editing. WG: Conceptualization, Writing – review & editing. SM: Conceptualization, Funding acquisition, Writing – review & editing. AP: Conceptualization, Funding acquisition, Project administration, Writing – review & editing.
